# Error analysis of a heating oven for a spin-exchange relaxation-free magnetometer

**DOI:** 10.1038/s41598-025-10457-5

**Published:** 2025-07-06

**Authors:** Min-Hwan Lee, Sanghyun Park, Younguk Ryu, Hyogi Kim, Hyun-Gue Hong, Geol Moon

**Affiliations:** 1 Department of Physics, Chonnam National University, Gwangju, 61186 Republic of Korea; 2 Center for Quantum Technologies, Chonnam National University, Gwangju, 61186 Republic of Korea; 3 Korea Research Institute of Standards and Science, Daejeon, 34113 Republic of Korea

**Keywords:** Characterization and analytical techniques, Quantum optics

## Abstract

We present the design and error analysis of a non-magnetic electric heating oven for spin-exchange relaxation-free (SERF) magnetometers, where precise thermal control and minimal magnetic disturbance are critical. A compact oven using a double-layer polyimide-constantan heating film was developed and evaluated through finite element simulations and experiments. Temperature simulations showed good agreement with measurements using a convective heat transfer coefficient of $7.6~\mathrm {W/m^2\cdot K}$. However, a temperature difference of $14.94~^\circ$C was observed due to heat loss near the vapor cell stem. Adding a thermal shell in the simulation reduced this gradient to $6.43~^\circ$C, indicating improved thermal uniformity. Magnetic field simulations initially showed large discrepancies with experimental results. Including a twisted wire pair improved agreement, but differences remained. To further investigate the remaining discrepancies, Monte Carlo simulations were performed by introducing realistic variations in fabrication tolerances, mounting positions, and wiring configurations. The results revealed that wiring errors had the greatest influence on the measured magnetic field. These findings provide key insights into structural factors affecting magnetic performance and offer practical guidelines for reducing magnetic noise in SERF magnetometer systems.

## Introduction

The spin-exchange relaxation-free (SERF) magnetometer is a highly sensitive, low-noise device used in life sciences^1^, earth sciences^2^, and fundamental research^3^. It achieves sensitivity below $1~\mathrm {fT/\sqrt{Hz}}$ by minimizing spin-exchange relaxation in high-density atomic and low magnetic field conditions^4^. To achieve high sensitivity, the implementation of the SERF regime is essential. In atomic magnetometers, the sensitivity is limited by the coherence time of the atomic ensemble, which is primarily disrupted by spin-exchange collisions^5^. However, it is well known that decoherence caused by spin-exchange collisions can be suppressed in the SERF regime^6,7^. This regime is realized when the spin-exchange rate greatly exceeds the Larmor precession frequency, such that atoms undergo many collisions during a single precession cycle and the collision effects are averaged out. To satisfy these conditions, both high atomic density and near-zero magnetic field environments are required. In SERF magnetometers, high atomic density is achieved by heating the vapor cell. Consequently, the heating structure becomes a key component that directly governs the magnetometer’s performance. Various heating methods have been developed to meet these operational requirements. Heating methods include hot air^8,9^, laser^10,11^, and electric heating^12,13^. Electric heating, preferred for its high power, precise temperature control, cost-efficiency, and potential for miniaturization, may induce magnetic fields that could impair magnetometer performance.

To minimize magnetic induction, design approaches include optimizing heating film structures via genetic algorithms^14,15^, using MEMS for structural precision and miniaturization^16^, and adding heating film layers to enhance magnetic field self-cancellation^17^. Despite these advances, mitigating induced magnetic fields in electric heating systems remains challenging. Discrepancies between simulation and experimental results pose a significant challenge in evaluating the effectiveness of magnetic field suppression strategies. Representative studies illustrate the extent of this issue. Liu et al.^14^ simulated a magnetic field of $1.7825~\mathrm {pT/mA}$ at the center of the vapor cell for a double-layer structure, while their experimental measurement using a fluxgate sensor reported $250.2~\mathrm {pT/mA}$ approximately 140 times higher. Lu et al.^15^ reported a simulated average magnetic field of $200~\mathrm {pT/mA}$ for their optimized structure, whereas atomic magnetometer measurements yielded $7.1~\mathrm {pT/mA}$ (*x*-axis), $-73.0 ~\mathrm {pT/mA}$ (*y*-axis), and $-46.8~\mathrm {pT/mA}$ (*z*-axis). The vector norm of these components is approximately $87.0~\mathrm {pT/mA}$, about 2.3 times lower than the simulated value. Liang et al.^17^ employed a quadra-layer design, simulating $0.3136~\mathrm {pT/mA}$ at the vapor cell center, while the experimentally measured volume-averaged magnetic field was $16.6~\mathrm {pT/mA}$, about 53 times higher. Zhou et al.^18^ simulated an average magnetic field of $0.392~\mathrm {nT/mA}$ inside the vapor cell for an optimized circular coil structure. Their measurements showed $0.523~\mathrm {nT/mA}$ (*x*-axis), $2.823~\mathrm {nT/mA}$ (*y*-axis), and $5.528~\mathrm {nT/mA}$ (*z*-axis), yielding a vector norm of approximately $6.23~\mathrm {nT/mA}$ about 16 times higher than the simulation. However, in many of these cases, direct comparison is complicated by inconsistencies in spatial domains and measurement approaches; for example, simulations often report the norm of the magnetic field, whereas experiments typically measure individual vector components. Thus, simple ratio comparisons should be interpreted with caution. In contrast, our proposed oven structure produced an average magnetic field of $12.8~\textrm{nT}$ ($25.6~\mathrm {pT/mA}$) inside the vapor cell under a heating current of $500~\textrm{mA}$ with twisted wires included, and $17.8964~\mathrm {pT/mA}$ at the center of the vapor cell. The experimentally measured value was approximately twice the simulated result, indicating that our design achieves competitive magnetic suppression performance (Table 1). Nonetheless, such mismatches are often attributed to multiple factors, including fabrication tolerances, asymmetric wire arrangements, mounting misalignments, differences in the sensitive volume between the sensor and simulation, and temporal variations in ambient magnetic fields. Most prior works have only addressed these sources qualitatively, lacking quantitative analysis. To address these limitations, our study incorporated several methodological improvements. First, we modeled the sensitive volume of the fluxgate sensor as an ideal cylindrical region to improve consistency between simulation and experiment. Second, we employed a through-hole via structure to ensure reliable electrical connections between heating film layers. Third, we incorporated the actual geometry of the current supply wires in the simulation model for a more accurate representation of field sources. Finally, we conducted a Monte Carlo-based statistical analysis to quantitatively evaluate the impact of structural errors on magnetic field induction. This integrated approach offers a robust framework for magnetic field mitigation and constitutes a meaningful advancement in the design of heating structures for SERF magnetometers.

## System design

**Fig. 1:**
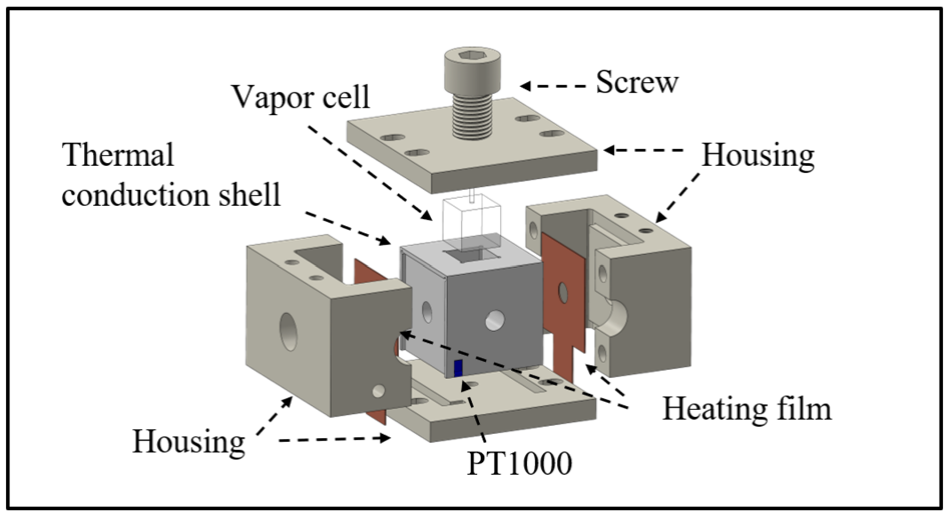
Schematic of the vapor-cell oven design.

Our heating oven consists of the following components: a boron nitride (BN)^19^ thermal conduction shell, which is nonmagnetic with a high thermal conductivity; a polyether ether ketone (PEEK)^20^ housing, which provides high heat resistance and strength for securing and insulating the components; a heating film consisting of polyimide^21^, which provides heat resistance and electrical insulation; and constantan^22^, which imparts high electrical and thermal resistance. Copper is used for through-hole vias and to form pads for the heating film’s electrical connections. Figure 1 illustrates the designed structure of the heating oven.

A $10\times 10\times 10~\mathrm {mm^{3}}$ vapor cell, with $1~\textrm{mm}$-thick walls and a $5~\textrm{mm}$-long conical stem, is centrally located in the oven. It is encased in a thermal conduction shell that ensures a fixed distance from the heating film and effective heat transfer. The shell measures $25\times 25\times 25~\mathrm {mm^{3}}$ and features cylindrical holes of $8~\textrm{mm}$ diameter intersecting at the center of each side. One side of the shell is open for vapor cell insertion and removal, and it has a recessed structure for mounting the heating film. The housing secures the heating film, PT1000 temperature sensor, and thermal conduction shell using PEEK screws. PEEK posts and holders adjust the oven’s height and position.

**Fig. 2:**
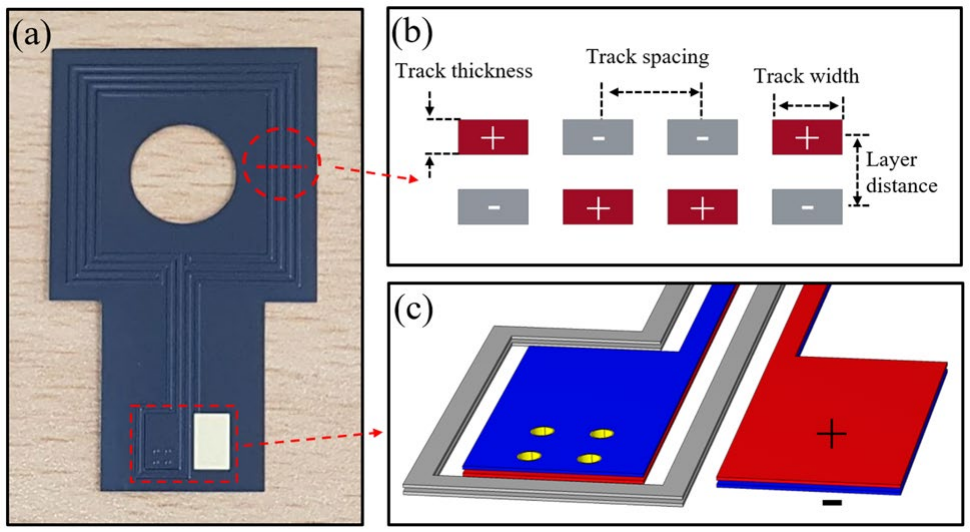
(**a**) Photograph of the electrical heating film; (**b**) current direction and geometric parameters of the heating film; (**c**) electrical polarity of individual layers (red and blue indicate positive and negative, respectively) and through-hole vias.

The induced magnetic field in electric heating is closely linked to the resistance track structure, necessitating careful selection. Liu et al.^14^ optimized the heating film structure using a genetic algorithm under various constraints, which guided our design. The resistance track’s structure parameters are defined by track spacing (s), track width (w), track thickness (t), and layer distance (d) (shown in Fig.2b); in this study, we used the values 0.8, 0.4, 0.05, and $0.112~\textrm{mm}$, respectively, for these parameters. These values were selected within the parameter range proposed in Ref.^12^, with careful consideration of both magnetic field self-cancellation performance and fabrication feasibility. For example, the values for spacing (s) and width (w) were chosen based on parameter combinations that minimized magnetic fields in prior optimization studies, while the thickness (t) and layer distance (d) reflect the actual physical dimensions of the polyimide insulation and adhesive layers employed. Some of these values lie near the lower bounds of our fabrication capabilities, indicating that further optimization may be achievable with improved manufacturing precision. A double-layered heating film was fabricated, as multilayer structures better self-cancel the magnetic field compared to single-layer structures^23^. Figure 2a shows the manufactured film’s black exterior due to a black polyimide coverlay. Stable electrical connections are essential for the multilayer structure. To control these variables, a through-hole via structure is introduced, ensuring consistent electrical connections within the heating film; the electrical connection structure is shown in Fig.2c. The measured resistance of a double-layered heating film is $16.16~\mathrm {\Omega }$.

## Simulation and experimental design

Finite element method-based simulations were conducted using the AC/DC and Heat Transfer Modules in COMSOL Multiphysics. Temperature simulations incorporated the Electric Currents, Heat Transfer in Solids, and Electromagnetic Heating physics interfaces, while magnetic field simulations utilized the Magnetic Fields physics interface. A user-controlled mesh was applied for standard simulations. All components except the heating film were discretized using extremely fine free tetrahedral elements. For the heating film, which consists of thin, high-aspect-ratio multilayers, an extremely fine 2D triangular mesh was generated on each surface and swept in the thickness direction to form a structured 3D mesh. In Monte Carlo simulations, a physics-controlled mesh was employed to reduce computational load, with a slightly relaxed resolution that still ensured sufficient accuracy. In temperature simulations, the ambient temperature was set to $20^{\circ }\textrm{C}$, and natural convection boundary conditions were applied to all external surfaces of the oven, including the top, sides, and bottom. The convective heat transfer coefficient was set in the range of 5 to $10~\mathrm {W/m^{2}\cdot K}$, based on standard values for free convection in air^24^. Radiative heat loss was not considered. The vapor cell was modeled as being filled with air at atmospheric pressure (1 atm). The built-in air properties provided by COMSOL Multiphysics were used without modification. Due to the small internal volume of the vapor cell ($10\times 10\times 10~\mathrm {mm^{3}}$) and a Rayleigh number below 1700^25^, natural convection inside the cell was neglected, and heat transfer within the cell was modeled as pure conduction.

For temperature measurements, a precision current source (Keithley 2400 SourceMeter) was used to heat the oven equipped with the vapor cell, and a PT1000 sensor was connected to a digital multimeter (Keithley DMM6500) using the RTD method to measure the temperature. Magnetic field measurements were conducted using a fluxgate magnetometer(Bartington MAG-01H, axial Mag Probe Type B). Since our study focuses on static magnetic fields induced by heating current, we did not use modulation techniques. All measurements were performed after reaching thermal and magnetic stability. To minimize external influences, the system was enclosed in a $\mu$-metal magnetic shield. For each condition, five repeated measurements were conducted, and the mean and standard deviation were calculated. To assess baseline uncertainty, background magnetic fields were measured continuously for 30 minutes without heating current. The average was $1.740~\textrm{nT}$ with a standard deviation of $0.213~\textrm{nT}$, which was treated as the system noise floor. The fluxgate probe used has a sensing coil length of approximately $28~\textrm{mm}$ and a known sensitive volume of $0.0023~\mathrm {cm^3}$. Due to this characteristic, the magnetic field measured in the experiment reflects an average magnetic field over the entire sensitive volume, rather than the value at a single point. Accordingly, in the simulation, this sensitive volume was approximated as an ideal cylindrical region and incorporated into the model, and the spatial average of the magnetic field within this volume was calculated to ensure consistency with the experimental result. Twisted wire pairs (silver-plated copper wires, core diameter: $0.3~\textrm{mm}$, jacket thickness: $0.175~\textrm{mm}$) with a pitch of $10~\textrm{mm}$ minimized magnetic induction.

## Result and analysis

### Temperature measurement and simulation analysis

Accurately determining the convective heat transfer coefficient (crucial for our heat-loss simulation model) is essential for matching actual temperature distributions. However, owing to its dependence on environmental factors like fluid velocity, temperature difference, and material properties, determining the convective heat transfer coefficient in complex systems is challenging. We compared temperature measurements with simulations to identify the optimal convective heat transfer coefficient and predict the temperature distribution inside the vapor cell oven.

**Fig. 3:**
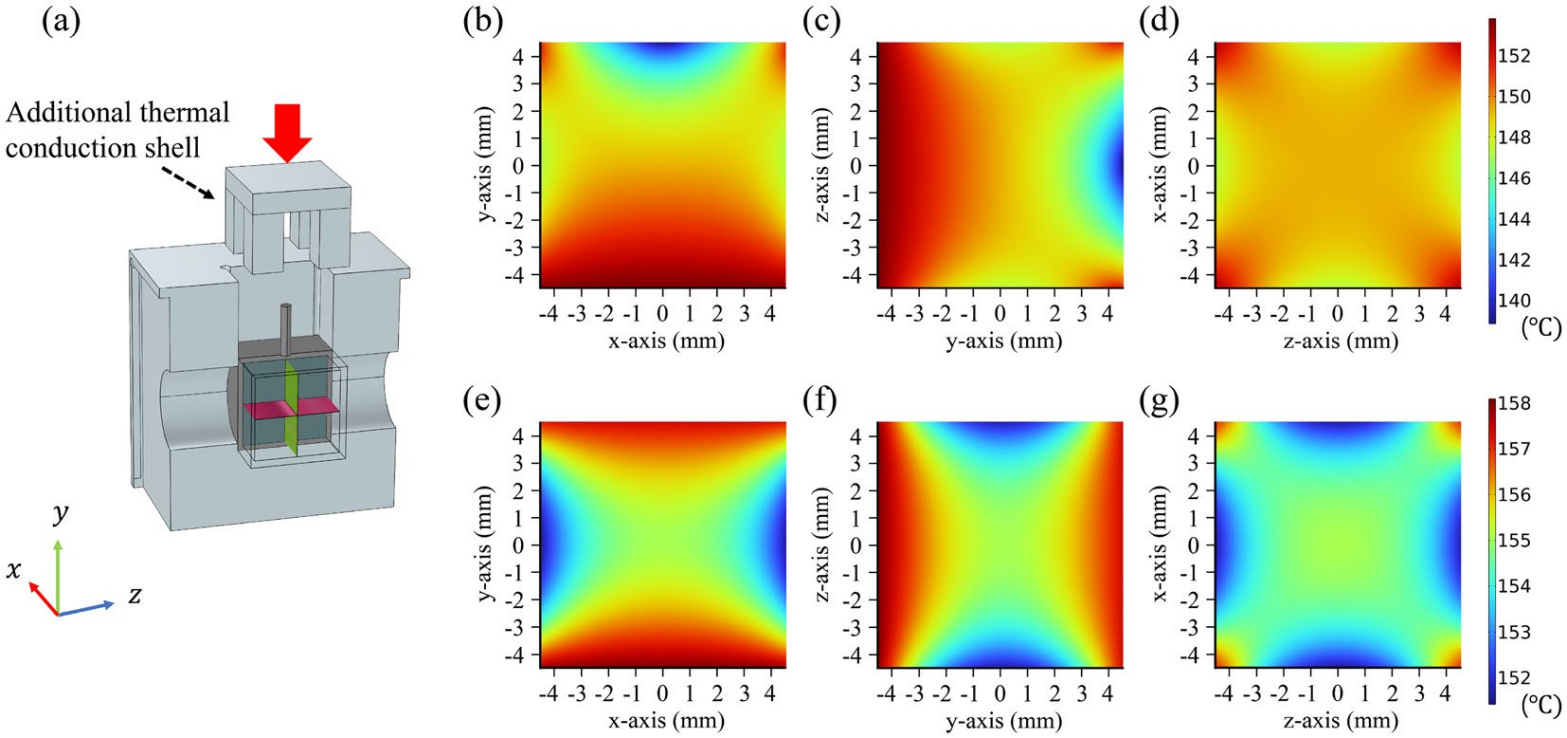
(**a**) Illustrations of the vapor cell mounted in the thermal conduction shell and the additional thermal conduction shell (Cross-sectional view). (**b**–**d**) Temperature distribution inside the vapor cell without an additional thermal conduction shell. (**e**–**g**) Temperature distribution after integrating an additional thermal conduction shell around the stem.

At a current of $0.5~\textrm{A}$, the PT1000 temperature sensor measured $153.61~^{\circ }\textrm{C}$. By adjusting the convective heat transfer coefficient in the simulation, we matched with the experimental result at $7.6\mathrm {W/m^{2}\cdot K}$. Using this coefficient, we predicted the temperature distribution inside the vapor cell (Fig.3b–d), with the figures showing the temperature distribution across planes centered on the vapor cell.

The average temperature inside the vapor cell is $150.00~^{\circ }\textrm{C}$, with maximum and minimum values of $153.81~^{\circ }\textrm{C}$ and $138.87~^{\circ }\textrm{C}$, respectively. The vapor cell experiences a temperature difference of about $14.94~^{\circ }\textrm{C}$, mainly due to heat loss at the stem. This occurs because the stem lacks direct contact with the thermal conduction shell, resulting in lower temperatures. This can cause differential thermal expansion, potentially damaging the vapor cell, and a temperature gradient, leading to nonuniform vapor density and increased common-mode noise in gradient magnetometers^26,27^. A simple and effective method to reduce this temperature distribution difference is to add a thermal conduction shell around the stem, thereby restricting convection in its vicinity and reducing heat loss. To evaluate the effectiveness of this solution, we conducted simulations by applying the additional thermal conduction shell shown in Fig. 3a. The temperature distribution obtained from these simulations is presented in Fig. 3e–g. The simulation results showed that when a current of $0.5~\textrm{A}$ was applied, the average temperature inside the vapor cell increased to $155.55~^{\circ }\textrm{C}$, and the temperature difference between the maximum and minimum values decreased to $6.43~^{\circ }\textrm{C}$. Additional simulations confirmed that attaching a thermal shell near the stem significantly improves temperature uniformity. The temperature difference $\Delta \textrm{T}$ within the vapor cell decreased from $14.94~^{\circ }\textrm{C}$ to $6.43~^{\circ }\textrm{C}$, mainly due to reduced thermal gradients near the stem. Although this structure was not experimentally implemented in the present work, the results suggest its feasibility and potential advantages. In future studies, we plan to fabricate the proposed structure and experimentally evaluate its performance under operating conditions. Simulations were also conducted to assess the accuracy of the PT1000 sensor in reflecting the vapor cell’s average temperature. At a current of $0.5~\textrm{A}$, the simulated average temperature difference between the vapor cell and PT1000 sensor was about $3.6~^{\circ }\textrm{C}$, with the sensor’s temperature slightly higher due to its proximity to the heating film. This small difference indicates the sensor reliably represents the cell’s internal temperature.

### Magnetic field measurement and simulation analysis

Magnetic field simulations are crucial for evaluating the performance of the heating oven in a SERF magnetometer. Accurate simulations ensure reliable assessments, but discrepancies can arise due to various sources of error. Most of these errors originate from the simplification of the actual experimental environment in the simulation model. Precisely replicating the experimental conditions in simulations is extremely challenging. As a result, certain elements must be simplified or omitted. However, if essential factors are excluded, significant discrepancies between actual performance and simulated performance may occur. Therefore, identifying these critical factors is a key step in enhancing simulation accuracy. By assessing the influence of various error sources and distinguishing between negligible and essential factors, it is possible to incorporate only the most significant elements into the simulation. In this section, we compare simulation results with experimental data to assess the discrepancies between measured and simulated values. Subsequently, in next section, we analyze the contributions of various sources of error through simulations.

**Fig. 4:**
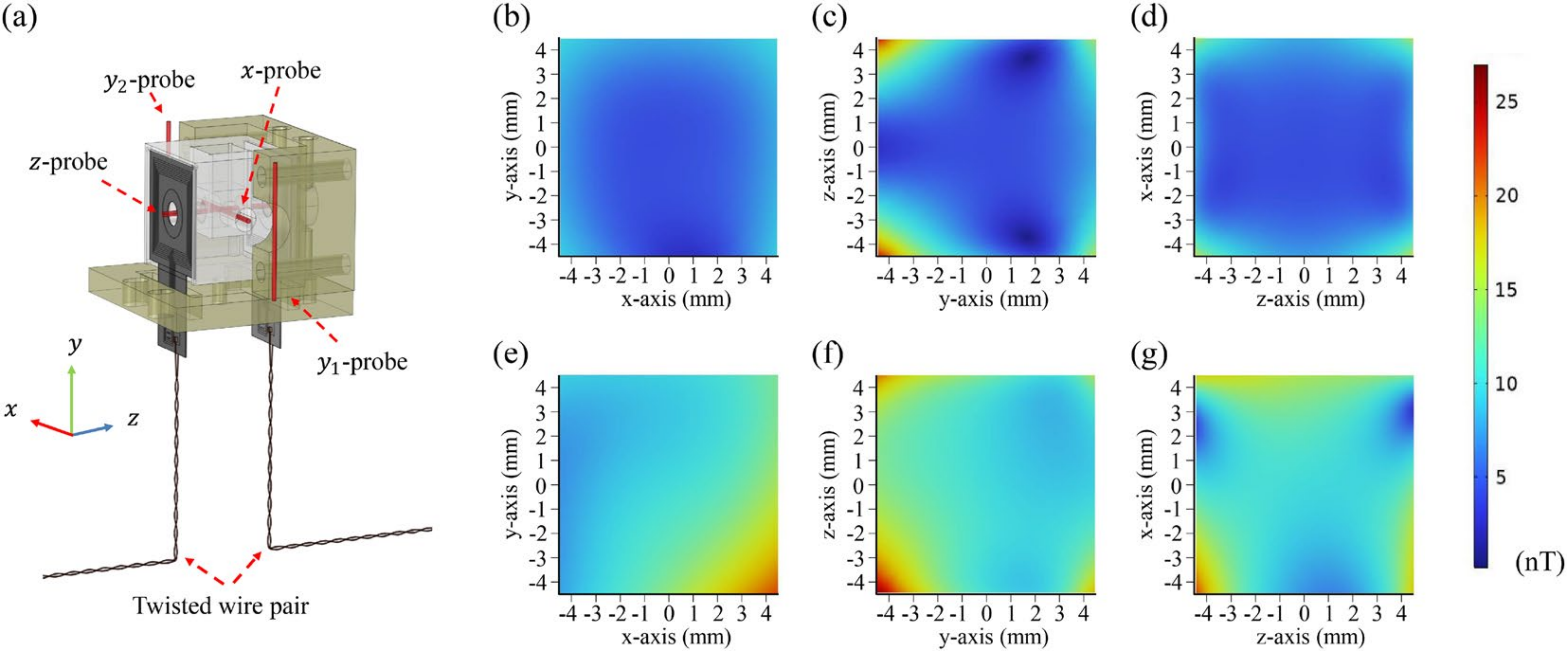
(**a**) Overall simulated structure with the twisted wire pair. (**b**–**d**) Distribution of the magnetic field magnitude in the ideal case without the twisted wire pair. (**e**–**g**) Distribution of the magnetic field magnitude with the twisted wire pair.

Figure 4a depicts the magnetic field simulation model, showing the coordinate axes and overall structure. The model includes a twisted wire pair bent at a $90~^{\circ }$ where it contacts the bottom surface. Additionally, this figure presents the positions (*x*-probe, $y_1$-probe, $y_2$-probe, *z*-probe). The *x*-probe and *z*-probe were directly inserted for measurement, whereas direct insertion along the *y*-axis was not possible due to physical obstruction from the oven structure. Instead, the $y_1$ and $y_2$ probes were placed on the outer surface of the housing to measure the magnetic field along the *y*-axis.

Simulation results showed that under a heating current of $0.5~\textrm{A}$, the average magnetic field magnitude inside the vapor cell without the twisted wire pair was $9.02~\textrm{nT}$, which increased by approximately $40\%$ (to $12.8~\textrm{nT}$) upon incorporating the twisted wire pair. This result indicates that the twisted wire pair significantly affects the magnetic field by increasing the field magnitude and asymmetrically altering its distribution. Figure 4b–g present the magnetic field distribution on planes centered at the vapor cell’s origin. The twisted wire pair, offset from the vapor cell centerline, causes an asymmetrical field distribution.

**Fig. 5:**
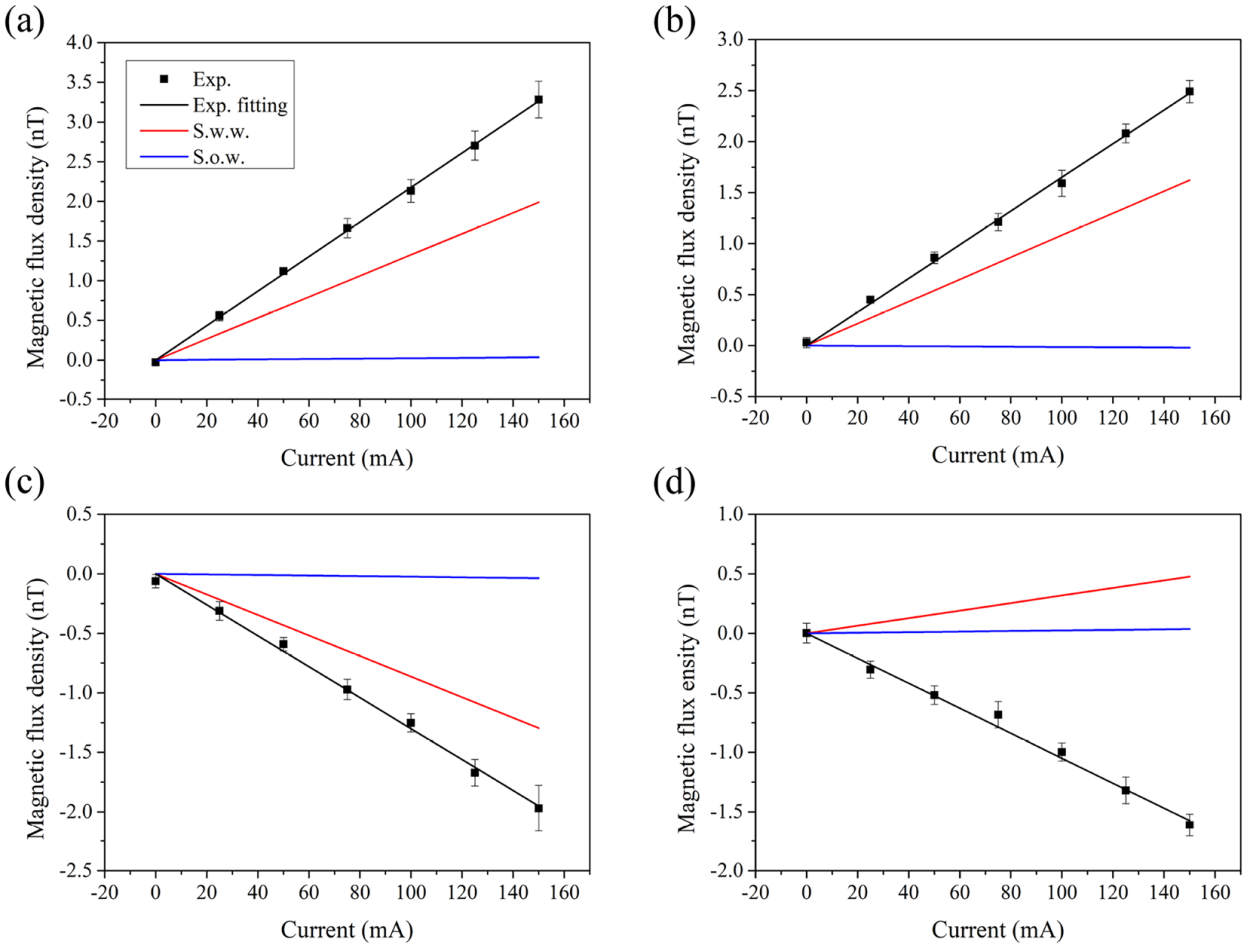
Comparison between measured and simulated magnetic flux density components at each probe position. The *x*-probe, $y_1$-probe, $y_2$-probe, and *z*-probe correspond to specific magnetic field measurement positions, as indicated in Fig. 4. (**a**) *x*-axis component at the *x*-probe. (**b**) *y*-axis component at the $y_1$-probe. (**c**) *y*-axis component at the $y_2$-probe. (**d**) *z*-axis component at the *z*-probe. In all panels, black markers indicate experimental measurements averaged over five repetitions. Red lines represent simulation results including the twisted wire pair, and blue lines represent results without the wires. The simulated values represent spatial averages within ideal cylindrical volumes matching the sensitive region of each probe.

We measured the magnetic field and compared the results with simulations as shown in Fig. 5. The measured values represent the average magnetic flux density in the probe’s active region, and the simulation calculated the same value for comparison. The measurements are offset-corrected. The comparison indicates that the simulation results with the twisted wire pair align more closely with the measurements at all probe positions. To quantitatively assess this agreement, we introduce a magnetic field gradient with respect to current, defined by the following equation:

1 $$\begin{aligned} B_{\text {axis}}=k_{\text {axis}}\times I-b \end{aligned}$$

where $B_{\text {axis}}$ represents the magnetic field (T) in each axial direction, $k_{\text {axis}}$ shows the gradient of the magnetic field with respect to the current *I* for each axis, and *b* denotes the magnetic field offset. The calculated gradient values are listed in Table1.

**Table 1:** $k_{\text {axis}}$ values for each probe.

Type	$k_x$	$k_{y_1}$	$k_{y_2}$	$k_z$
Exp.	22.30	16.21	-12.53	-10.37
S.w.w.	13.27	10.82	-8.64	3.18
S.o.w.	0.16	-0.13	-0.24	0.33

Exp.: Experimental result; S.w.w.: Simulation with twisted wire pair; S.o.w.: Simulation without wires. All units are in $\mathrm {pT/mA}$.

Table 1 compares experimental and simulation results for each probe, showing that the ratios of absolute values between simulation and experimental results are on average approximately $\sim 84$ without and $\sim 2$ with the twisted wire pair. Although including the current supply configuration improves simulation accuracy, significant discrepancies persist between experimental and simulation results. Further simulations with structural modifications were conducted to identify additional potential error sources.

### Monte Carlo-based error analysis of magnetic field discrepancies

**Fig. 6:**
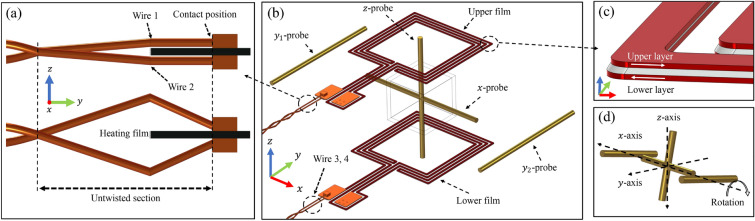
Schematic illustrations of the directions and locations of error sources considered in Monte Carlo simulations. (**a**) Schematic illustrating the wiring error. The variable untwisted_length represents the length of the untwisted section; negative values indicate an increase in length. Contact# (where # symbol denotes wire index 1 to 4) represents the displacement of the connection positions (wire 1–4) along the *x*-axis. wire#_ *z* and wire#_ *x* describe the position deviation of the center of the untwisted section along the *z*- and *x*-axes, respectively. The figure compares the ideal configuration with a case in which wire1_ *z* and wire2_ *z* are both shifted by $0.5~\textrm{mm}$. (**b**) The full FEM simulation model with the actual positions of the magnetic field measurement probes. The positions of the probes (*x*-probe, $y_{1}$-probe, $y_{2}$-probe, and *z*-probe) are shown as configured in the experiment. These probe locations serve as reference points for comparing the simulated magnetic field with experimental results. Additionally, the positions of the upper and lower heating films are indicated in the model. (**c**) Schematic illustrating the fabrication tolerance category, focusing on alignment errors between the upper and lower heating film layers. Positional offset in the *x*- and *y*-directions are defined as *x* _offset_upper, *y* _offset_upper, *x* _offset_lower, and *y* _offset_lower, while track_rotation represents the rotational misalignment between the two layers. (**d**) Schematic illustrating the mounting error category, which includes positional and angular deviations of the magnetic field probes. Each probe may exhibit translational errors along its axial and off-axis directions (probe_axial_pos,probe_offaxis_pos), as well as rotational misalignments about the *x*-, *y*-, and *z*-axes (probe_rot_ *x* , probe_rot_ *y* , probe_rot_ *z* ). All illustrations in this figure are schematic representations and not to scale.

In the previous section, incorporating the twisted wire pair into the magnetic field simulation significantly improved agreement with experimental results. However, full consistency across all probe positions was not achieved, particularly for the $k_z$ component. This indicates that additional structural uncertainties, beyond the basic current supply configuration, may contribute to the remaining discrepancies. To investigate this further, we performed a comprehensive Monte Carlo simulation to statistically evaluate the influence of key error sources on the magnetic field distribution. Potential structural deviations were categorized into three groups: *mounting error*, *fabrication tolerance*, and *wiring error*. For each category, a set of physically realistic tolerance ranges was defined based on assembly and manufacturing constraints. Within these ranges, input parameters were randomly sampled using a uniform distribution, a common choice when no prior statistical distribution is known. Each Monte Carlo simulation consisted of 1000 realizations per category, with the magnetic field gradients ($k_x$, $k_{y1}$, $k_{y2}$, and $k_z$) and the circuit resistance evaluated in every run. Based on the calculated resistance values, simulations were screened to identify electrically faulty configurations, which were excluded from statistical analysis. In our simulations the circuit resistance of a properly formed structure typically ranged from $14.22~\mathrm {\Omega }$ to $18.11~\mathrm {\Omega }$. Only simulations with an average element quality of the mesh above 0.57 were used for statistical analysis. Table 2 lists the input variables and their tolerance ranges used in the Monte Carlo simulations and Fig. 6 illustrates their physical configurations along with the coordinate axes for each error category in more detail.

**Table 2:** List of input variables and their tolerance ranges used in the Monte Carlo simulations.

Category	Variable Name	Range	Unit	Description
Mounting Error	probe_axial_pos	±3	mm	Axial position error of the probe
	probe_offaxis_pos	±1	mm	Off-axial position error of the probe
	probe_rot_ *x*	±2	deg	Rotation error around *x*-axis
	probe_rot_ *y*	±2	deg	Rotation error around *y*-axis
	probe_rot_ *z*	±2	deg	Rotation error around *z*-axis
	film_rot_upper	±2	deg	Rotation error of upper film around *z*-axis
	film_rot_lower	±2	deg	Rotation error of lower film around *z*-axis
	film_ *x* _upper	±2	mm	Mounting error of upper film in *x* direction
	film_ *y* _upper	±2	mm	Mounting error of upper film in *y* direction
	film_ *z* _upper	±1	mm	Mounting error of upper film in *z* direction
	film_ *x* _lower	±2	mm	Mounting error of lower film in *x* direction
	film_ *y* _lower	±2	mm	Mounting error of lower film in *y* direction
	film_ *z* _lower	±1	mm	Mounting error of lower film in *z* direction
Fabrication Tolerance	track_spacing_error	±0.02	mm	Track spacing error
	track_width_error	±0.01	mm	Track width error
	track_thickness_error	±0.005	mm	Track thickness error
	layer_gap_error	±0.01	mm	Layer distance error
	*x* _offset_upper	±0.1	mm	*x* offset of upper track layout center
	*y* _offset_upper	±0.1	mm	*y* offset of upper track layout center
	*x* _offset_lower	±0.1	mm	*x* offset of lower track layout center
	*y* _offset_lower	±0.1	mm	*y* offset of lower track layout center
	track_rotation	±0.1	deg	Rotation error of track around *z*-axis
Wiring Error	untwisted_length	±1	mm	Length variation of the untwisted section
	wire1_ *z*	0 to 0.5	mm	*z* position error of wire 1
	wire1_ *x*	$-0.3$ to 0.5	mm	*x* position error of wire 1
	wire2_ *z*	0 to 0.5	mm	*z* position error of wire 2
	wire2_ *x*	$-0.3$ to 0.5	mm	*x* position error of wire 2
	wire3_ *z*	0 to 0.5	mm	*z* position error of wire 3
	wire3_ *x*	$-0.3$ to 0.5	mm	*x* position error of wire 3
	wire4_ *z*	0 to 0.5	mm	*z* position error of wire 4
	wire4_ *x*	$-0.3$ to 0.5	mm	*x* position error of wire 4
	contact1	±0.5	mm	Contact position error of wire 1 (*x*-axis)
	contact2	±0.5	mm	Contact position error of wire 2 (*x*-axis)
	contact3	±0.5	mm	Contact position error of wire 3 (*x*-axis)
	contact4	±0.5	mm	Contact position error of wire 4 (*x*-axis)

The variables are categorized into mounting errors, fabrication tolerances, and wiring errors. Each variable was sampled within the defined range using a uniform distribution.

**Fig. 7:**
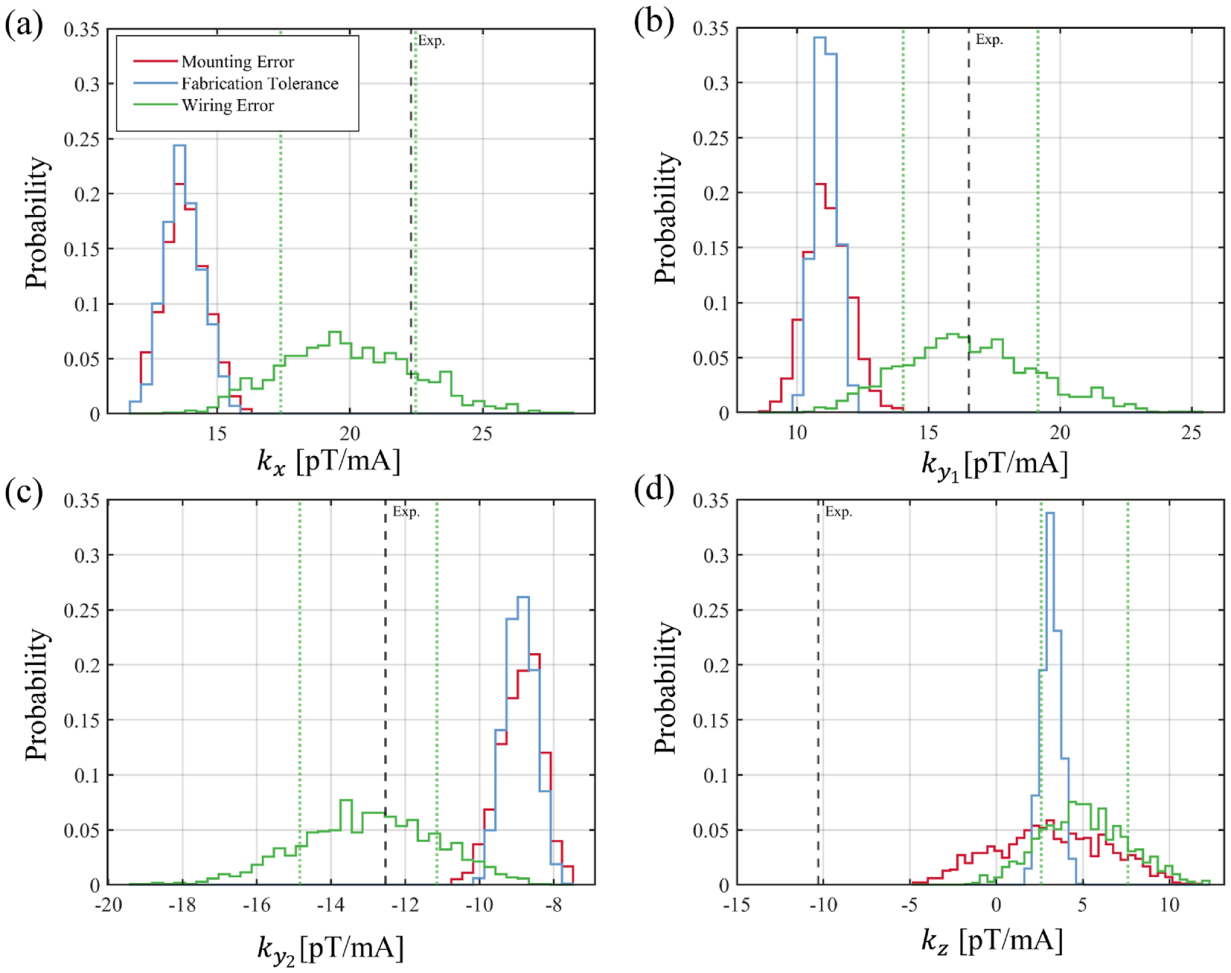
Monte Carlo simulation results for the magnetic field gradient components ($k_{x}$, $k_{y_1}$, $k_{y_2}$, and $k_{z}$) under three categories of error: mounting error, fabrication tolerance, and wiring error. For each category, 1000 simulations were performed, and the results were visualized as probability-normalized histograms. The black dashed lines indicate the experimentally measured values, while the green dashed lines represent the $\pm 1\sigma$ range from the wiring error simulations.

Figure 7 shows the statistical distribution of each gradient component for the three error categories. For the $k_x$, $k_{y1}$, and $k_{y2}$ components, the results indicate that the wiring error category exhibits the broadest distribution, and only this category encompasses the experimentally measured values within the $\pm 1\sigma$ range of the simulations. In contrast, the mounting error and fabrication tolerance categories yield narrower distributions that fail to explain the experimental observations for these components. In the fabrication tolerance category, the Pearson correlation coefficients between the track structure parameters (track_spacing_error, track_width_error, track_thickness_error, and layer_gap_error) and the magnetic field gradient components were found to be very low. In our simulation model, each track structure parameter was applied uniformly to all resistance tracks. In practice, fabrication errors typically occur locally, such as at individual segments or corners of the track, but modeling such localized errors in finite element simulations is computationally intensive and geometrically complex. Therefore, to maintain modeling feasibility, we applied each parameter variation globally and identically across the entire track layout. This modeling assumption may partly explain the weak statistical correlation observed, as local asymmetries and nonlinear current pathways, which contribute to magnetic field distortion, were not fully represented in the simulation.

The situation differs significantly for the $k_z$ component. The experimental value of $k_z = -10.37~\mathrm {pT/mA}$ lies well outside the distribution ranges predicted by all three error categories. While the wiring and mounting error categories exhibit broader spreads in $k_z$ than fabrication tolerance, the experimental result remains unmatched. This discrepancy suggests that analyzing each error category independently may fail to capture the combined effects of multiple error sources, indicating the need for an integrated simulation approach that considers interactions across categories.

**Fig. 8:**
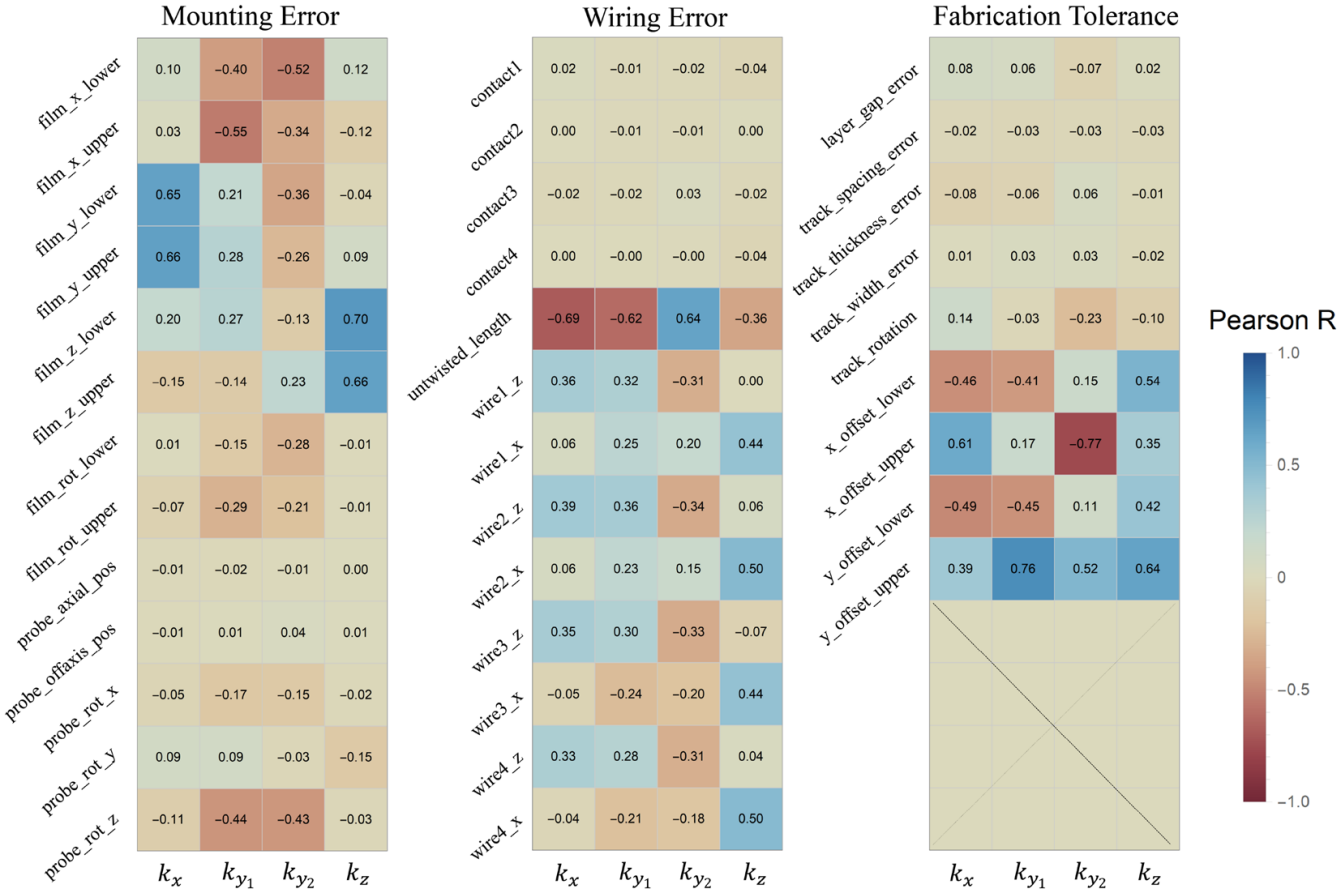
Heatmap of the Pearson correlation coefficients between each input variable ( categorized into mounting error, wiring error, and fabrication tolerance ) and the magnetic field gradient components ($k_{x}$, $k_{y_1}$, $k_{y_2}$, and $k_{z}$). The color scale represents the sign and magnitude of the correlation coefficients: positive correlations are shown in blue, and negative correlations in red. Larger absolute values of the coefficients indicate that the corresponding variable has a greater influence on changes in the magnetic field gradient.

To further analyze the sensitivity of the magnetic field gradients to input variables, we calculated Pearson correlation coefficients between each parameter and the gradient components. As shown in Fig. 8, parameters such as film_ *z* _upper and film_ *z* _lower (mounting error) and wire#_ *x* (wiring error) display strong correlations with the $k_z$ component while exhibiting weak or negligible influence on the other components. These results indicate that $k_z$ is uniquely susceptible to vertical misalignments of the heating film and lateral offsets in the untwisted wire section.

In our experimental setup, the current supply was deliberately designed to minimize the length of the untwisted section of the twisted wire pair, as shorter untwisted lengths reduce residual magnetic fields. However, this design choice imposes practical limitations. Due to mechanical tension and structural constraints, it is difficult to precisely align the two conductors in such a confined space. Consequently, physical deviations in the untwisted section are inevitable and represent a likely source of uncertainty in the $k_z$ measurement. In particular, wire#_ *x* variables, which strongly influence $k_z$, may have experienced misalignments that were not fully captured in the simulation, where their displacement range was limited to $-0.3~\textrm{mm}$ to $0.5~\textrm{mm}$ to avoid wire overlap. In reality, insulated wires can shift beyond this range due to installation flexibility, further contributing to the observed discrepancy.

**Fig. 9:**
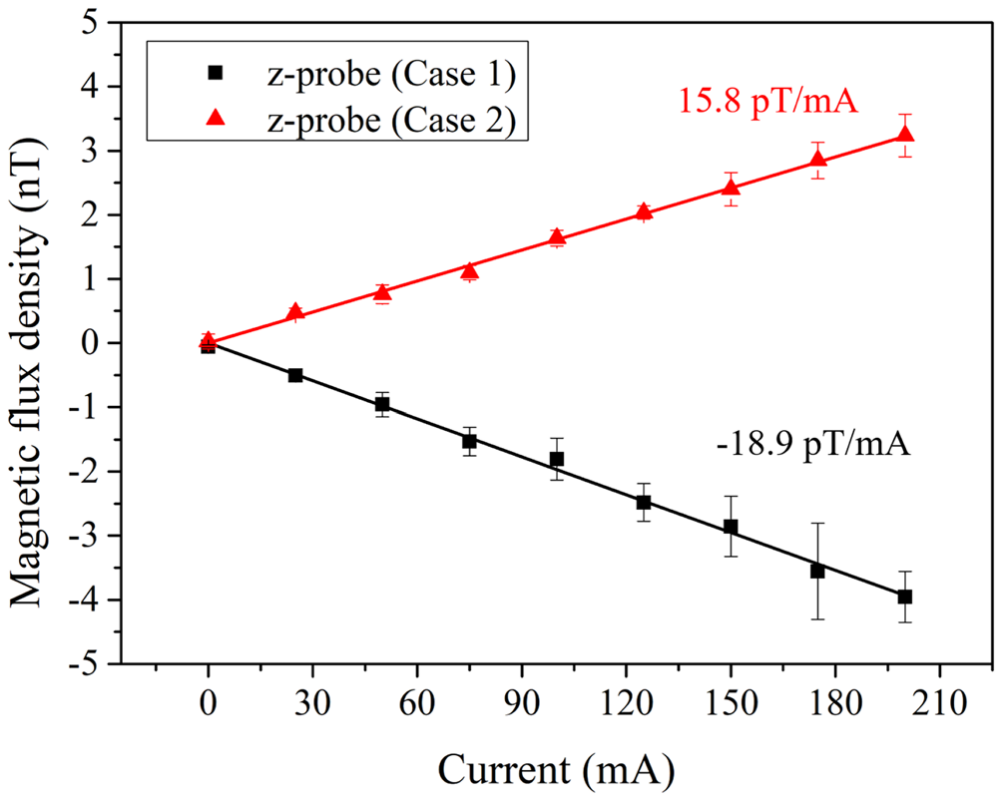
Measured magnetic field gradients at the *z*-probe under two different wire alignment configurations. In Case 1, the untwisted section of the wires is aligned along the *z*-axis, resulting in a negative gradient of $-18.9~\mathrm {pT/mA}$. In Case 2, the same wires are aligned along the *x*-axis, yielding a positive gradient of $+15.8~\mathrm {pT/mA}$. The reversal in both sign and magnitude of the $k_{z}$ component under nominally identical current and heating conditions highlights the extreme sensitivity of the vertical magnetic field gradient to the physical orientation of the untwisted wire segment. These results provide experimental validation for the conclusion that even small deviations in the wiring layout (particularly lateral offsets) can dominate the magnetic field behavior in SERF magnetometer systems.

To qualitatively validate this hypothesis, an additional experiment was performed in which the alignment direction of the supply wires was deliberately varied. As shown in Fig. 9, the $k_z$ component changed not only in magnitude but also in sign depending on whether the wires were aligned along the *z*-axis (Case 1: $-18.9~\mathrm {pT/mA}$) or the *x*-axis (Case 2: $+15.8~\mathrm {pT/mA}$). These results demonstrate that even under nominally identical conditions, the orientation of the untwisted wire segment can significantly alter the vertical magnetic field gradient. While this test exaggerates the untwisted length for visibility, it provides direct evidence that wire configuration critically affects the $k_z$ component.

In summary, the analysis reveals that the discrepancies in the $k_x$, $k_{y1}$, and $k_{y2}$ components can be primarily attributed to uncertainties in the wiring configuration. However, for the $k_z$ component, a more complex picture emerges. Its strong sensitivity to both vertical film alignment and lateral wire position suggests that no single error category is sufficient to explain the observed behavior. Instead, the $k_z$ discrepancy likely arises from a coupled effect of mounting and wiring errors, which is not captured in the current simulation framework. This limitation highlights the need for future modeling approaches that account for interactions among variables across different error categories, as well as extended tolerance bounds, to more fully reflect three-dimensional uncertainties related to structure in magnetically sensitive systems.

## Conclusion

This study presents a quantitative analysis of the thermal and magnetic characteristics of a non-magnetic electric heating oven for spin-exchange relaxation-free (SERF) magnetometers, based on both finite element simulations and experimental validation. The results identify key structural factors that significantly influence the performance of high-sensitivity magnetic field measurements. In particular, magnetic field simulations incorporating twisted wire pairs significantly improved agreement with experimental results. However, a notable discrepancy remained in the vertical magnetic field gradient component, $k_{z}$. Monte Carlo simulations, categorized into fabrication tolerances, mounting errors, and wiring errors, showed that while most gradient components could be explained by the wiring error category, $k_{z}$ could not be attributed to any single source. Pearson correlation analysis revealed that $k_{z}$ is highly sensitive to both the vertical alignment of the heating film and the lateral position of the untwisted wire segment. These parameters exhibited strong correlations, and their combination was identified as a potential major contributor to magnetic field distortion based on simulation results. Additional experiments confirmed this sensitivity, as merely changing the wire orientation reversed the sign of $k_{z}$, providing qualitative support for the simulation-based interpretation. In addition, thermal simulations showed that the introduction of a thermal conduction shell around the vapor cell stem significantly reduced internal temperature gradients, which can improve vapor density uniformity and reduce noise in magnetometer operation. Based on these findings, we propose the following design considerations for the future development of SERF oven systems:

- *First*, the current supply wires should be routed with sufficient separation from the vapor cell and follow well-defined geometrical paths. In particular, *untwisted wire segments* should not be placed near the heating film, even over short distances, to minimize induced magnetic fields.
- *Second*, the wire configuration must be mechanically secured after installation. Lateral displacement of the wires can reverse the direction of $k_{z}$, and changes in wire geometry after field nulling can compromise magnetic stability. Physical fixation using bonding or epoxy is therefore recommended.
- *Third*, a reference structure for aligning the heating film is necessary. The vertical position and rotational alignment of the film are critical factors affecting $k_{z}$, and appropriate alignment guides or fixtures should be incorporated.
- *Fourth*, a comprehensive simulation framework that accounts for the interaction between multiple error sources is essential. Single-variable models are insufficient for robust design; future systems should be evaluated using multivariate, statistically-driven tolerance analyses.

These thermal, magnetic, and statistical insights provide a practical foundation for optimizing the structural design of high-precision quantum sensing components. Future work will focus on the experimental implementation of the proposed improvements and the development of advanced simulation frameworks that incorporate correlated uncertainties, with the aim of enhancing the accuracy and reliability of SERF magnetometer systems.

## Data Availability

The datasets used and/or analyzed during the current study are available from the corresponding author upon reasonable request.
